# Atomoxetine Enhances Connectivity of Prefrontal Networks in Parkinson's Disease

**DOI:** 10.1038/npp.2016.18

**Published:** 2016-03-02

**Authors:** Robin J Borchert, Timothy Rittman, Luca Passamonti, Zheng Ye, Saber Sami, Simon P Jones, Cristina Nombela, Patricia Vázquez Rodríguez, Deniz Vatansever, Charlotte L Rae, Laura E Hughes, Trevor W Robbins, James B Rowe

**Affiliations:** 1 Department of Clinical Neurosciences, University of Cambridge, Cambridge, UK; 2 National Research Council, Institute of Bioimaging and Molecular Physiology, Catanzaro, Italy; 3 Key Laboratory of Mental Health, Institute of Psychology, Chinese Academy of Sciences, Beijing, China; 4 Systems and Automatic Control Engineering, Technical University of Cartagena, Cartagena, Spain; 5 Division of Anaesthesia, University of Cambridge, Cambridge, UK; 6 Sackler Centre for Consciousness Science, University of Sussex, Brighton, UK; 7 Department of Psychiatry, Brighton and Sussex Medical School, Brighton, UK; 8 MRC Cognition and Brain Sciences Unit, University of Cambridge, Cambridge, UK; 9 University of Cambridge Behavioural and Clinical Neuroscience Institute, Cambridge, UK

## Abstract

Cognitive impairment is common in Parkinson's disease (PD), but often not improved by dopaminergic treatment. New treatment strategies targeting other neurotransmitter deficits are therefore of growing interest. Imaging the brain at rest (‘task-free') provides the opportunity to examine the impact of a candidate drug on many of the brain networks that underpin cognition, while minimizing task-related performance confounds. We test this approach using atomoxetine, a selective noradrenaline reuptake inhibitor that modulates the prefrontal cortical activity and can facilitate some executive functions and response inhibition. Thirty-three patients with idiopathic PD underwent task-free fMRI. Patients were scanned twice in a double-blind, placebo-controlled crossover design, following either placebo or 40-mg oral atomoxetine. Seventy-six controls were scanned once without medication to provide normative data. Seed-based correlation analyses were used to measure changes in functional connectivity, with the right inferior frontal gyrus (IFG) a critical region for executive function. Patients on placebo had reduced connectivity relative to controls from right IFG to dorsal anterior cingulate cortex and to left IFG and dorsolateral prefrontal cortex. Atomoxetine increased connectivity from the right IFG to the dorsal anterior cingulate. In addition, the atomoxetine-induced change in connectivity from right IFG to dorsolateral prefrontal cortex was proportional to the change in verbal fluency, a simple index of executive function. The results support the hypothesis that atomoxetine may restore prefrontal networks related to executive functions. We suggest that task-free imaging can support translational pharmacological studies of new drug therapies and provide evidence for engagement of the relevant neurocognitive systems.

## Introduction

Parkinson's disease (PD) impairs cognition, including executive functions, attentional control, decision-making, verbal fluency, and response inhibition. These deficits may be present soon after diagnosis and impair quality of life despite dopaminergic therapy (Watson and Leverenz, 2010; Yarnall *et al*, 2013). New approaches are required to maintain or restore cognitive function, in addition to the mainstay of dopaminergic therapy.

Dopaminergic therapy does not satisfactorily restore executive functions in PD (Weintraub *et al*, 2006; Rowe *et al*, 2008), due in part to deficits in non-dopaminergic systems. For example, noradrenergic projections from the locus coeruleus to the cortex are severely affected by PD pathology (Braak *et al*, 2003). Loss of noradrenaline has been implicated in diverse deficits in executive functions such as inhibition, attention, working memory, cognitive flexibility, and verbal fluency (Ye *et al*, 2015; Kehagia *et al*, 2014; Vazey and Aston-Jones, 2012; Williams-Gray *et al*, 2007; Robbins and Arnsten, 2009). Noradrenergic drugs therefore provide a potential mechanism to restore cognitive functions in selected patients (Kehagia *et al*, 2014; Ye *et al*, 2015).

Noradrenergic projections from the locus coeruleus reach the inferior frontal gyrus (IFG), presupplementary motor area (pre-SMA), dorsolateral prefrontal cortex, and anterior cingulate cortex (Goldstein *et al*, 2011). These are critical areas for executive function, inhibition, working memory, and fluency (Gauthier *et al*, 2009). For example, the right IFG and pre-SMA are important for response inhibition and attentional processing (Rae *et al*, 2015; Sharp *et al*, 2010) and are modulated by noradrenaline (Ye *et al*, 2015; Vazey and Aston-Jones, 2012).

The selective noradrenaline reuptake inhibitor atomoxetine increases noradrenergic neurotransmission within the prefrontal cortex (Bymaster *et al*, 2002). In preclinical studies, atomoxetine improves attentional set-shifting (Newman *et al*, 2008) and response inhibition (Robinson *et al*, 2008). Atomoxetine also improves response inhibition and increases right IFG activation during inhibition by healthy humans (Chamberlain *et al*, 2009).

Preliminary trials of PD have shown that atomoxetine can improve inhibition and attention in some patients (Kehagia *et al*, 2014; Ye *et al*, 2015). Using task-based functional magnetic resonance imaging (fMRI), Ye *et al* (2015, 2016) showed that the behavioral effect of atomoxetine on inhibitory control in Parkinson's patients was related to enhanced right IFG activity and stronger frontostriatal connectivity.

However, the majority of pharmacological imaging studies, including Ye *et al* (2015, 2016), use task-based paradigms. This approach has several potential disadvantages including performance confounds, practice effects across sessions, and the ambiguity arising from activation differences in the context of very different performance by patients (Price and Friston, 1999). They also require participant training and are limited to the relatively narrow range of neural systems related to the task.

There is an alternative approach, using task-free fMRI (also known as resting-state fMRI). This minimizes training demands, task-related confounds, and practice effects across sessions. It also allows for inclusion of cognitively and physically impaired patients. In the absence of a task, one cannot study task-related activations but one can examine changes in brain network connectivity.

We therefore used task-free fMRI to investigate the effect of atomoxetine on brain function in patients with PD, using a double-blinded, randomized placebo-controlled crossover design. We measured functional connectivity of the right IFG because of this region's central role in executive function, including inhibition and fluency, and in mediating the behavioral benefits of atomoxetine (Levy and Wagner, 2011). We used seed-based correlation methods to compare functional connectivity of the right IFG, first contrasting patients on placebo with controls (between-group design) and then contrasting patients on placebo to patients on atomoxetine (within-group design). Individual differences were accommodated using covariates of age, drug plasma concentration, disease severity, and the change in out-of-scanner cognitive performance under atomoxetine *vs* placebo.

We tested the specific hypotheses that (i) PD reduces functional connectivity of the right IFG with other regions implicated in executive function including the pre-SMA, dorsolateral prefrontal, and anterior cingulate cortex; and (ii) atomoxetine restores the functional connectivity of the right IFG to these regions.

## Materials and methods

### Participants

Thirty-three people with idiopathic PD were recruited from the Cambridge University PD Research Clinic using UK PD Society Brain Bank criteria. Subsets of these patients have been included in previous studies reporting data sets from functional tasks (Ye *et al*, 2015, 2016). Seventy-six healthy age- and sex-matched controls were recruited from the Medical Research Council's Cognition and Brain Sciences Unit volunteer panel and healthy volunteers registered with the Cambridge University PD Research Clinic, at the John van Geest Centre for Brain Repair. Inclusion criteria were (1) age between 45 and 80 years; (2) non-demented, with Mini-Mental State Examination (MMSE) >26/30, noting that this does not exclude mild cognitive impairment; and (3) no significant current depression. None of the patients reported symptoms or behaviors of impulse control disorders at interview.

Participants underwent assessment with the MMSE, digit span forward and backward, category and letter fluency (Rittman *et al*, 2013) plus the revised Beck Depression Inventory. Patients were assessed with the Unified PD Rating Scale motor subscale III at the start of each session. All participants provided written informed consent. The study was approved by the local research ethics committee and was exempted from clinical trials status by the United Kingdom Medicines for Human use Regulatory Agency.

We anticipated that any potential future use of noradrenergic drugs for cognition would be adjunctive to dopaminergic medication, not a replacement. Therefore, all patients were tested on their regular medication, to assess the effect of atomoxetine in the context of clinically optimized standard dopaminergic and/or cholinergic therapy. Levodopa equivalent dose (LED) was calculated for each patient (Tomlinson *et al*, 2010). Participant demographics and clinical characteristics are summarized in Table 1.

### Experimental Design

The study used a double-blinded placebo-controlled crossover design for patient treatment by atomoxetine and placebo, randomized within successive blocks of six recruits to maintain balanced groups. Each patient underwent two separate sessions of cognitive and neurological assessments and brain imaging, at least 6 days apart but at approximately the same time of day on each session. At the start of each session patients received a 40 mg oral dose of atomoxetine or placebo. They were transferred to the MRI suite 2 h after the drug administration to coincide with the peak plasma concentration of atomoxetine (Sauer *et al*, 2005: unpublished day-curve data in a separate group of 20 PD patients confirms peak plasma levels between 120 and 180 min). Control participants were scanned once without the drug to provide normative data. The principal analysis is of the main effect of drug treatment within PD, not a drug by group interaction.

### fMRI Data Acquisition and Preprocessing

Task-free functional imaging was performed at rest using a TIM-Trio 3T Magnetic Resonance Imaging (MRI) scanner (Siemens Medical Systems, Erlangen, Germany). A minimum of 145 volumes was acquired using an echo-planar imaging (EPI) sequence (repetition time (TR) 2000 ms, echo time (TE) 30 ms, matrix=64 × 64, in-plane resolution of 3 × 3 mm, 32 slices of 3 mm thickness with a 0.75 mm interslice gap, and a flip angle (FA) of 78°). Structural Magnetization-Prepared Rapid Acquisition with Gradient Echo (MPRAGE) scans (TR of 2300 ms, TE of 2.86 ms, matrix=192 × 192, in-plane resolution of 1.25 × 1.25 mm, 144 slices of 1.25 mm thickness, inversion time of 900 ms and FA of 9°) were also acquired during the same session.

We used a preprocessing pipeline optimized for older subjects to take into account atrophy and subject's head movement in the scanner (Patel *et al*, 2014). The Diffeomorphic Anatomical Registration Through Exponentiated Lie Algebra (DARTEL) algorithm (Ashburner, 2007) created a study-specific template from MPRAGE images. Unified six-tissue class segmentation was applied to structural images and grey and white matter segments from all participants were warped together iteratively over six steps to create a study-specific template, which was then affine transformed to MNI space.

We used a customized version of the brainwavelet toolbox (www.brainwavelet.org) to perform preprocessing of functional images. The first five volumes were removed and the mean EPI image was coregistered to the T1 image and then transformed to MNI space using the flow fields generated during the DARTEL processing. Subsequent processing of the functional time series included slice-timing correction to correct for acquisition delay, combined regression of cerebrospinal fluid (CSF) signal and motion derivatives, high-pass band filter (0.01 Hz), and wavelet despiking (Patel *et al*, 2014). Spatial smoothing was applied with an 8 mm isotropic Gaussian kernel.

In-scanner head movements can produce spurious correlations in task-free fMRI data (Power *et al*, 2012). In the current study, we combined several approaches to minimize these motion-related effects on the blood oxygen level-dependent (BOLD) signal. First, the wavelet despiking toolbox was used during preprocessing: movements related to non-stationary events in each voxel were identified and despiked from the time course. Second, six parameters of head motion were used as regressors during seed-correlation analysis (see Functional connectivity analysis section below). Third, four participants were excluded (one control, three patients) based on a high average root mean-squared (RMS) displacement computed from the translation parameters of head motion: average RMS displacement over 2 SDs from the mean and/or 2 SDs from the mean difference between placebo and atomoxetine sessions.

We also assessed motion between imaging sessions within the patient group. The average RMS displacement did not differ significantly between placebo and atomoxetine sessions (*p*=0.79, *t*=0.26). It is therefore unlikely that drug effects on measures of functional connectivity were driven by cross-session differences in head movement.

### Functional Connectivity Analysis

To investigate the effect of disease and drug on functional connectivity between brain regions, seed-to-voxel connectivity maps were created for each subject per condition. Statistical Parametric Mapping software (SPM12, http://www.fil.ion.ucl.ac.uk/spm) was used to perform seed-based correlation functional connectivity analysis. A seed was created for the right IFG as a 5 mm radius sphere (MNI coordinates: 56, 16, and 12). The coordinates for this seed region were derived from a separate data set acquired by Ye *et al* (2015). The time series of the seed region was extracted from the rs-fMRI data for each individual. Signals deriving from the CSF and white matter (WM) were extracted using template masks.

The mean time series of the seed region was then correlated with the time series of each voxel in the whole brain in a multiple regression model. Head motion parameters as well as WM and CSF signals were included as nuisance regressors to minimize the influence of these non-neuronal signals on correlations with the IFG seed region. The voxel-wise parameter estimates of linear regression were used to create correlation maps for each subject and for each session, which were then Fisher z-transformed to correct the variance in the distribution of correlation coefficients. Individual correlation maps were used in a second-level general linear model to compare functional connectivity of the right IFG between controls and patients (on placebo) and to determine if the drug-enhanced connectivity in patients on atomoxetine *vs* placebo. Two-sample *t*-test maps were thresholded at *p*<0.05 voxel-level FWE-whole brain-corrected plus an exploratory analysis at threshold *p*<0.001 uncorrected.

Age, LED, drug plasma concentration, UPDRS-III, and the change in neuropsychological performance (eg: fluency) between atomoxetine and placebo sessions were included as covariates to investigate the influence of individual differences on treatment response.

## Results

### Participant Demographics

Data from 75 controls and 30 patients were used in the final analysis. A summary of demographic and clinical measures for controls and patients is reported in Table 1. Patients and healthy controls were matched in terms of sex, age, and education. PD subjects had lower MMSE and category fluency scores compared with controls as expected.

### Right IFG Connectivity Between Controls and Patients

Comparing controls and patients on placebo revealed a reduction in functional connectivity between the right IFG and left IFG/dorsolateral prefrontal cortex as well as the left cerebellum (Table 2 and Supplementary Figure S1). Regions with reduced functional connectivity between the right IFG at the exploratory threshold also included the dorsal anterior cingulate and pre-SMA.

### The Effect of Atomoxetine on Connectivity

**Figure 1:**
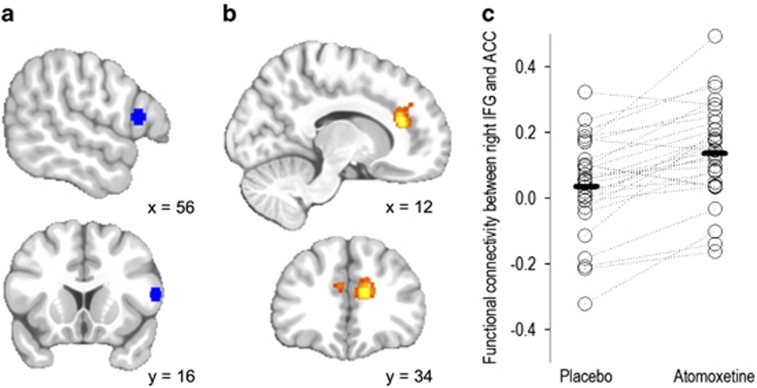
Whole-brain seed-correlation comparing right inferior frontal gyrus (IFG) connectivity between placebo and atomoxetine sessions in patients with Parkinson's disease. (a) Right IFG seed region centered on 56, 16, and 12 (in blue). (b) Voxel-wise correlation map showing increased connectivity after atomoxetine in patients, between right IFG and bilateral dorsal anterior cingulate (in orange) (atomoxetine>placebo; peak *p*<0.05 family-wise error (FWE)-corrected, *z*=4.73). (c) Scatter plot showing the functional connectivity between right IFG and dorsal anterior cingulate peak during placebo and atomoxetine sessions for each patient. ACC, anterior cingulate cortex.

Atomoxetine increased the functional connectivity between the right IFG and dorsal anterior cingulate in PD patients (*p*<0.05 FWE-corrected, see Figure 1 and Table 2). The dorsal anterior cingulate region with increased connectivity with the right IFG was bilateral but asymmetrical, mainly on the right side. Age, LED, drug plasma concentration, and UPDRS-III did not significantly interact with the drug-induced changes in connectivity.

**Figure 2:**
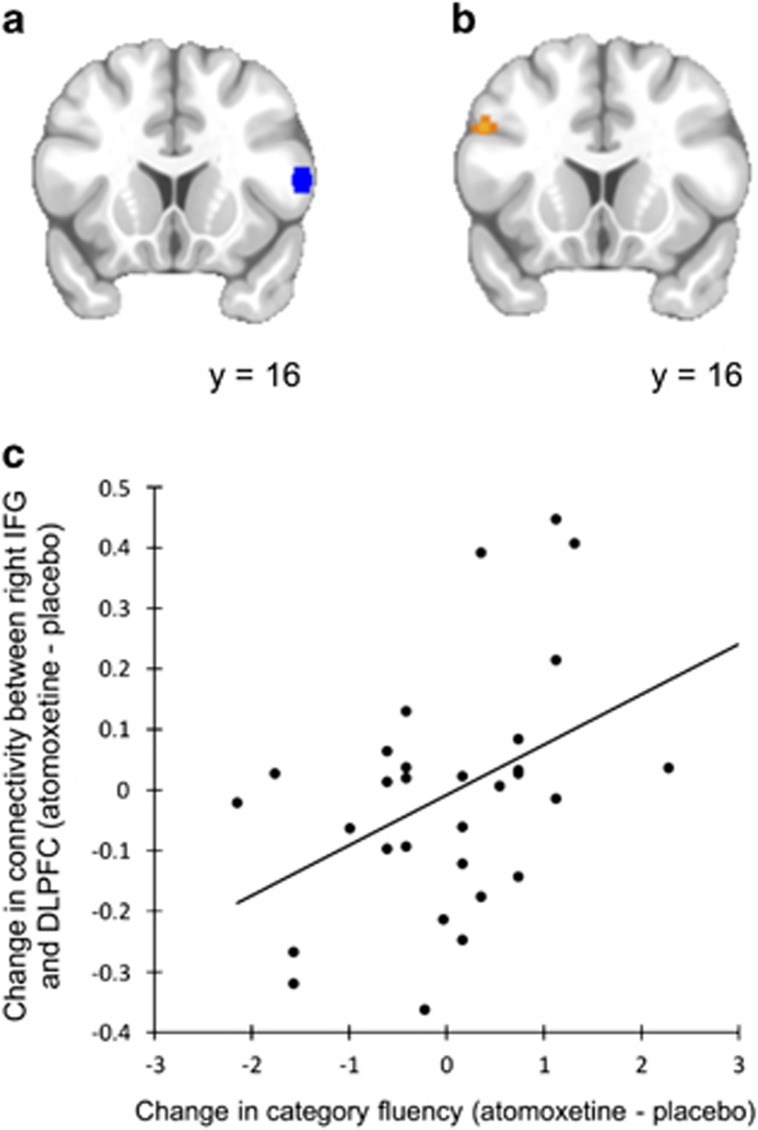
Seed-correlation comparing connectivity between placebo and atomoxetine sessions in patients with Parkinson's disease. (a) The seed region in right inferior frontal gyrus (IFG) (in blue). (b and c) Altered connectivity between the right IFG and left dorsolateral prefrontal cortex (DLPFC) on atomoxetine compared with placebo interacted with the change in category fluency between these two sessions (in orange). Patients who improved on the verbal fluency task while on atomoxetine also demonstrated increased connectivity between these regions (*p*=0.001 uncorrected).

There was an interaction between the effect of atomoxetine on right IFG connectivity and category fluency during each session. Specifically, patients with greater improvement in their out-of-scanner category fluency immediately before imaging (on atomoxetine relative to placebo) also demonstrated greater increases in functional connectivity between the right IFG and the left dorsolateral prefrontal cortex (see Figure 2 and Table 2). There was no interaction between drug plasma concentration, or any other covariate, and the effect of atomoxetine on right IFG functional connectivity.

## Discussion

We have shown that the selective noradrenergic reuptake inhibitor atomoxetine increases the functional connectivity between the right IFG and dorsal anterior cingulate cortex in PD. These two regions form a critical network for executive function, as evidenced by studies of task-based fMRI and focal brain lesions, but their interaction was revealed in this study by task-free fMRI. Atomoxetine also increased the connectivity between the IFG and dorsolateral prefrontal cortex, in proportion to the change in executive function (indexed by verbal fluency). Previous task-based investigations have indicated noradrenergic enhancement of regional activity and/or functional connectivity in the frontal lobe (Ye *et al*, 2015; Cubillo *et al*, 2014; Chamberlain *et al*, 2009). However, the ability to detect homologous modulations of network connectivity in the resting state, without complex tasks, opens up the use of fMRI to examine candidate therapeutic strategies for cognitive enhancement with reduced demands on timing constraints, training, and performance confounds.

The reduction in task-free connectivity correlates with both cognitive decline (Olde Dubbelink *et al*, 2014) and neuropsychiatric complications (Yao *et al*, 2014) and abnormal connectivity in PD has previously been reported (Kwak *et al*, 2010). In our study, patients showed reduced connectivity compared with healthy controls, between right IFG and the left IFG/dorsolateral prefrontal cortex and dorsal anterior cingulate. This accords with task-based studies of connectivity (Ye *et al*, 2015) and cognitive performance (Kehagia *et al*, 2014).

Taken together, these results support the neurobiological model of noradrenergic regulation of executive functions (Robbins and Arnsten, 2009), based on connectivity within the medial and lateral prefrontal cortex. Atomoxetine increased connectivity in this network, on a whole-group level. These patient data are consistent with the preclinical evidence. For example, enhanced noradrenergic neurotransmission in the medial prefrontal cortex facilitates attentional set-shifting performance in rats (Lapiz and Morilak, 2006), whereas noradrenergic depletion impairs attention during distracting conditions (Carli *et al*, 1983). Human imaging studies also suggest that interactions between dorsal anterior cingulate and right IFG support the executive function of attentional control and inhibition. For example, they are active during low-frequency events and error trials in choice discrimination and response inhibition tasks (Braver *et al*, 2001); during performance monitoring and attentional control (Botvinick *et al*, 2004); and in mediating response strategies during inhibition via the pre-SMA and subcortical structures (Sharp *et al*, 2010; Rae *et al*, 2015). The right IFG was the only seed region included in the analysis, because of task-based evidence of its involvement in executive dysfunction in PD and the neural mechanisms mediating the effect of atomoxetine on cognition. However, it is possible that atomoxetine also acts on other regions/networks and that these are also relevant to the drugs' cognitive benefits.

Given the integration of these regions within networks regulating executive control, we speculate that atomoxetine increased causal connections between them, known as effective connectivity. However, a seed-based correlational analysis does not provide direct evidence for effective integration. The analysis of effective connectivity is facilitated by strong anatomical priors and a task-based design, even where stochastic dynamic causal models are used to study task-free networks. It complements task-free methods that were the focus of this study in view of their advantages in terms of training, performance, and generalization to multiple networks.

The partial restoration of noradrenergic function in PD patients significantly modulated right IFG-left dorsolateral prefrontal cortex connectivity as a function of atomoxetine-induced changes in verbal fluency (category fluency task). In other words, people showing the highest degree of improvement in verbal fluency were also those who displayed the greatest effect of atomoxetine on functional connectivity within the prefrontal cortex. This result may explain the heterogeneity of drug response in PD and suggests that task-free fMRI might be used to develop biomarkers of individual differences modulating response to pharmacological treatments targeting cognition. Verbal fluency is impaired in PD (Williams-Gray *et al*, 2007) and we hypothesized that this was related to noradrenergic and structural deficits in prefrontal networks (Goldstein *et al*, 2011; Rae *et al*, 2012). The dorsolateral prefrontal cortex has also been implicated in verbal fluency (Frith *et al*, 1991; Cuenod *et al*, 1995) and noradrenergic transmission in this region is important for other executive functions (Arnsten, 2011). In PD patients, transcranial direct current stimulation of the dorsolateral prefrontal cortex improved verbal fluency and enhanced functional connectivity in verbal fluency networks (Pereira *et al*, 2013). The right IFG is active during verbal fluency tasks (Gaillard *et al*, 2000) and lesions to this region impair category fluency (Biesbroek *et al*, 2015). Therefore, we suggest that the functional connectivity between the right IFG and left dorsolateral prefrontal cortex represents the integration of executive strategies to support verbal category fluency.

There are several limitations to the current study. First, our patients received a single dose of atomoxetine, in conjunction with the brain imaging. However, it is possible that changes in functional connectivity following chronic drug administration are different (Koda *et al*, 2010). Homeostatic regulation, including for example, downregulation of noradrenergic receptors or synthesis, may ameliorate the effects of the drug. In practice, this could be offset by dose escalation, and in chronic therapy, patients with PD have tolerated up to 100 mg daily (Marsh *et al*, 2009). Nonetheless, future trials of clinical efficacy would need to assess longer-term treatment. Noradrenergic neurons in the LC exhibit both tonic and phasic responses, and our data alone do not discriminate the impact of atomoxetine on them. However, preclinical data indicate that atomoxetine increases the phasic-to-tonic firing ratio of the LC, thereby enhancing noradrenaline release in the frontal cortex (Florin-Lechner *et al*, 1996). We speculate that this effect mediates these changes in functional connectivity. However, the neurochemical mechanisms underlying the effect of atomoxetine may not be exclusively related to its effect on noradrenergic transmission. For example, atomoxetine may act via noradrenergic systems alone or together with dopaminergic transmission (Bymaster *et al*, 2002). Whereas animal studies suggest that the benefits of atomoxetine on cognitive control are mediated primarily by noradrenergic systems (Bari *et al*, 2009), interactions with dopamine cannot be excluded. For example, connectivity between the lateral and medial prefrontal cortex is partially dopamine dependent in the context of motor control (Rowe *et al*, 2010), and cognitive control is enhanced by drugs with joint dopaminergic and noradrenergic effects such as methylphenidate (Moeller *et al*, 2014). It should also be noted that dopaminergic drugs can modulate BOLD signal fluctuations (Kwak *et al*, 2012); however, this variable was kept constant in the within-subject analysis comparing PD patients on and off atomoxetine.

This study is not a clinical trial, and ultimately any new therapy would be judged by its clinical impact, on cognition, behavior, or motor control. However, we have demonstrated that the modulation of functional connectivity in prefrontal regions by atomoxetine in the task-free state is concordant with understandings of networks relevant to executive function as well as with changes in performance on neuropsychological assessments performed outside the scanner. This is in line with the correspondence between brain networks identified in task-free and task-based imaging (Smith *et al*, 2009). This technique also offers insight into how individual differences influence treatment response in terms of one or more brain networks, implicitly demonstrating target engagement in the central nervous system while minimizing the difficulties in learning or executing challenging cognitive tasks. We suggest that this technique is a useful contributor to understanding drug effects on the brain, especially in early stages of translation, but it would be a prelude to rather than a substitute for clinical trials.

In conclusion, this study supports the noradrenergic hypothesis for frontal lobe function and, indirectly, its role in cognition. We demonstrate that task-free fMRI can be used to examine therapies targeting cognitive systems and investigate individual differences associated with treatment response. Further work is needed for optimization of potential noradrenergic therapies for PD, and to establish the limits of this approach more generally in defining the impact of drugs on neurocognitive systems.

## Funding and disclosure

This work was funded by the Wellcome trust (103838), Parkinson's UK, National Institute for Health Research's Cambridge Biomedical Research Centre, and the Medical Research Council (MC-A060-5PQ30 and RG62761) and the James F McDonnell Foundation (twenty-first century science initiative on Understanding Human Cognition). The BCNI is supported by a joint award from the Wellcome Trust and Medical Research Council.

## Figures and Tables

**Table 1:** Participant Clinical and Demographic Characteristics

	**PD patients**, **mean (SD)**	**Controls**, **mean (SD)**	**Difference (*p*-value)**
Male:female	19 : 11	41 : 34	NS
Age (years)	67 (7.3)	67.1 (8.4)	NS
Education (years)	14.2 (3.6)	14.8 (4.0)	NS
MMSE	28.4 (1.7)	29.2 (1.1)	0.009
Category fluency	18.3 (5.5)	24.3 (6.2)	0.0001
Letter fluency	16.0 (4.4)	18.3 (5.7)	NS
Digit span forward	7.0 (1.1)	7.3 (0.8)	NS
Digit span backward	5.5 (1.2)	6.0 (1.3)	NS
Disease duration (years)	10.5 (4.4)	—	—
LED (mg per day)	870 (469)	—	—
UPDRS III ‘on'	22.6 (6.8)	—	—
Atomoxetine plasma concentration (ng/ml)	372.1 (167.4)	—	—

Abbreviations: LED, levodopa equivalent dose; MMSE, Mini-Mental State Examination; NS, nonsignificant; PD, Parkinson's disease; UPDRS III, the Unified Parkinson's disease rating scale motor subscale.

Groups are compared by unpaired *t*-test or *χ*^2^ test as appropriate (NS, *p*>0.05 uncorrected). LED according to the formula of Tomlinson *et al* (2010).

**Table 2:** Regions with Altered Connectivity with the Right Inferior Frontal Gyrus Seed Region (Controls *vs* Patients and Atomoxetine *versus* Placebo)

	**Coordinates**	***Z*-score**	**Cluster size (voxels)**
*Region with reduced connectivity in Parkinson's disease*			
Left IFG/dorsolateral prefrontal cortex	−44 6 24	4.73	2669
Dorsal anterior cingulate	10 42 32	3.68*	48
Pre-SMA	−10 2 56	3.85*	217
Cerebellum	−10 −54 −8	5.22	3376
			
*Region with increased connectivity on atomoxetine*			
Dorsal anterior cingulate (main effect of drug)	12 34 18	4.73	416
Left dorsolateral prefrontal cortex (drug interaction with fluency)	−50 20 34	3.98*	52

Abbreviations: DARTEL, Diffeomorphic Anatomical Registration Through Exponentiated L; IFG, inferior frontal gyrus; MNI, Montreal Neurological Institute; SMA, supplementary motor area.

Results are reported at whole brain family-wise error correction *p*<0.05, or as indicated by asterisk also at the exploratory threshold uncorrected *p*<0.001. Coordinates refer to the local peak in MNI space using a normalized study-specific template (DARTEL).
