# Supplementary figures and images for: Atomoxetine Enhances Connectivity of Prefrontal Networks in Parkinson's Disease
Source: Neuropsychopharmacology. 2016 Mar 2;41(8):2171–7. doi: 10.1038/npp.2016.18 (PMC4856878; doi:10.1038/npp.2016.18)

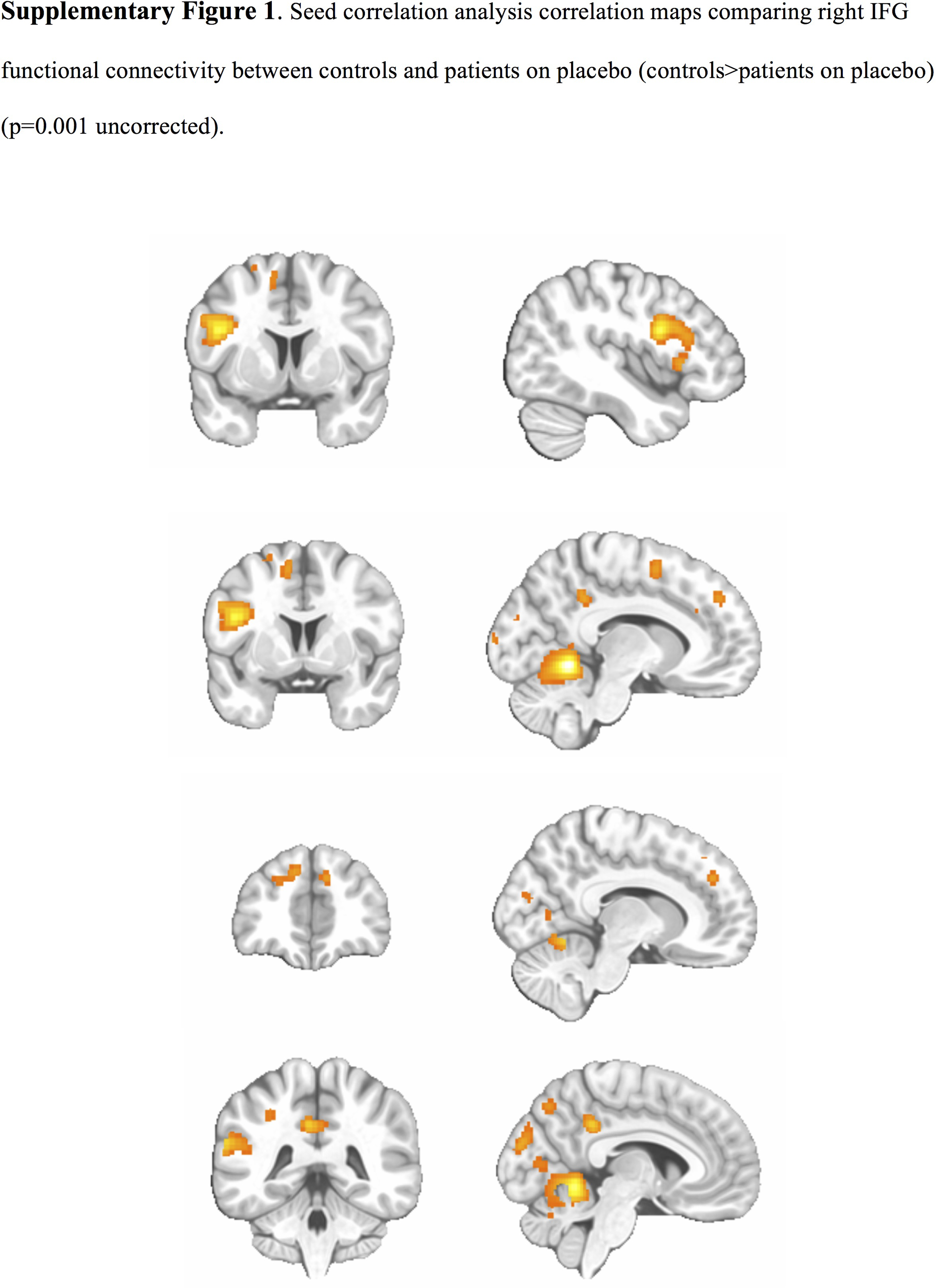

Supplement: Supplementary Figure S1 [file npp201618x1.tif]
